# *Schistosoma mansoni* Infections in Young Children: When Are Schistosome Antigens in Urine, Eggs in Stool and Antibodies to Eggs First Detectable?

**DOI:** 10.1371/journal.pntd.0000938

**Published:** 2011-01-04

**Authors:** J. Russell Stothard, Jose C. Sousa-Figuereido, Martha Betson, Moses Adriko, Moses Arinaitwe, Candia Rowell, Fred Besiyge, Narcis B. Kabatereine

**Affiliations:** 1 Wolfson Wellcome Biomedical Laboratories, Department of Zoology, Natural History Museum, London, United Kingdom; 2 Department of Infectious and Tropical Diseases, London School of Hygiene and Tropical Medicine, London, United Kingdom; 3 Vector Control Division, Ministry of Health, Kampala, Uganda; University of Maryland School of Medicine, United States of America

## Abstract

**Background:**

In Uganda, control of intestinal schistosomiasis with preventive chemotherapy is typically focused towards treatment of school-aged children; the needs of younger children are presently being investigated as in lakeshore communities very young children can be infected. In the context of future epidemiological monitoring, we sought to compare the detection thresholds of available diagnostic tools for *Schistosoma mansoni* and estimate a likely age of first infection for these children.

**Methods and Findings:**

A total of 242 infants and preschool children (134 boys and 108 girls, mean age 2.9 years, minimum 5 months and maximum 5 years) were examined from Bugoigo, a well-known disease endemic village on Lake Albert. Schistosome antigens in urine, eggs in stool and host antibodies to eggs were inspected to reveal a general prevalence of 47.5% (CI_95_ 41.1–54.0%), as ascertained by a positive criterion from at least one diagnostic method. Although children as young as 6 months old could be found infected, the average age of infected children was between 3¼–3¾ years, when diagnostic techniques became broadly congruent.

**Conclusion:**

Whilst different assays have particular (dis)advantages, direct detection of eggs in stool was least sensitive having a temporal lag behind antigen and antibody methods. Setting precisely a general age of first infection is problematic but if present Ugandan policies continue, a large proportion of infected children could wait up to 3–4 years before receiving first medication. To better tailor treatment needs for this younger ageclass, we suggest that the circulating cathodic antigen urine dipstick method to be used as an epidemiological indicator.

## Author Summary

In sub-Saharan Africa, intestinal schistosomiasis is a debilitating disease caused by a worm infection. To arrest disease progression, de-worming medications are given out, often *en masse*, to school-aged children. In Uganda, however, much younger children can be infected, and in lakeshore communities both infants and pre-school children can already show signs and symptoms of intestinal schistosomiasis. To change de-worming practices, further information on the occurrence of infections in these younger is needed for evidence-based decision making. Our study applied current methods of disease diagnosis to better define the ‘age of first infection’ and estimate general infection prevalence within a disease-endemic village. Up to 50% of young children were clearly shown to have schistosomiasis and could likely wait up to 3–4 years before obtaining first treatment if present de-worming policies are not changed. In the context of identifying future treatment needs, we propose that antigen detection methods are most suitable.

## Introduction

Throughout the last decade several large-scale preventive chemotherapy campaigns, waged against neglected tropical diseases, have progressively scaled up operations to reach nationwide coverage levels in Uganda [1], [2]. For control of intestinal schistosomiasis, as caused by *Schistosoma mansoni* infection, an active monitoring and surveillance programme, set within the national control programme (NCP), has provided important disease-specific information, assessing the impact of treatment upon the recipient population, as well as, re-alignment of original control objectives first set forth in 2003 [3], [4].

Following WHO guidelines, mass-drug administration of praziquantel (PZQ) is typically focused towards treatment of school-aged children (≥6 years) and adults who reside within disease endemic regions [5], [6]. PZQ is provided free of charge by the NCP and analysis of school and(or) community treatment registers has shown that several million people have received at least one annual treatment of PZQ within the last five years [1], [7]. Although this represents a considerable achievement, targeted epidemiological surveys have revealed that coverage is incomplete as in certain areas, e.g. shoreline environments of Lakes Victoria and Albert, large numbers of preschool-aged children (≤5 years) and infants (≤1 years) are infected with *S. mansoni* and have been largely overlooked by the treatment campaign [8], [9], [10].

To ensure that this unfortunate health inequality does not persist the treatment needs of younger children are being assessed and we have recently called for formal inclusion of these young children within the Ugandan NCP [11]. It can be safely assumed, for example, that mass-treatment initiatives are vital in most in shoreline villages where infections can be common. Given the geographical focality of schistosomiasis and itinerancy of lakeshore communities, however, an important future challenge for the NCP is collection of sufficient disease-specific information to better tailor local drug needs and set parameters for subsequent programme monitoring [12], [13]. Attention will therefore focus upon those sections of villages where young children are frequently bathed in freshly drawn lake water or are within range of regular ambulation to the lake margins.

Owing to the unique natural history and developmental biology of schistosomes within the mammalian host [14], accurate identification of infected cases is challenging [15], even more so in the younger child where the founding worm population has only recently established and begun to mature. Before female worms develop their full egg-laying capacity, sporadic deposition of eggs may take place with a proportion of these being voided into the bowel lumen and ejected in faeces whilst the remainder become trapped within the host's tissues [16]. Interacting with this are also the beginnings of the child's innate and adaptive immune responses to excretory-secretory products of the worms themselves, as well as these responses being primed or modulated by maternally induced effects, for example, during pregnancy and(or) breastfeeding [17], [18], [19], [20]. It is also of particular note that the child's immune system is in a maturing flux of recognition between self- and non-self epitopes [21] and the efficacy of PZQ, which is poor against immature worms of *S. mansoni* [22], is only starting to be explored in this ageclass [11]. From a general diagnostic perspective as existing tools are sub-optimal, improvement of methods and techniques for detection of intestinal schistosomiasis continues [15] but in the context of the younger child, it is not yet clear which of the present methods, or combinations thereof, is either most appropriate or applicable for routine use within the NCP.

We therefore report on a field-based study which attempted to determine the age of first infection in very young children with available techniques and also estimate, as accurately as possible, the general prevalence of intestinal schistosomiasis within this ageclass from a typical lakeshore community. The performance of methods that detect schistosome - antigens in urine, antibodies to egg antigens in serum and eggs in stool - was compared. For ease of comparison, our methods are subsequently referred to as: an antigen detection method (ADM), an indirect egg detection method (IEDM) and a direct egg detection method (DEDM), respectively.

## Materials and Methods

This field study was carried out in April 2009 in Bugoigo on Lake Albert (GPS co-ordinates, N 01° 54′.481″, E 31° 24′.597″), a fishing village impoverished both in terms of sanitation and hygiene that has been the location of several previous research/control studies on intestinal schistosomiasis [23], [24], [25], [26]. Prevalence of infection within local school-aged children has been continuously high (>50%) despite annual chemotherapy [27] and infections in infants and preschool children were first formally recorded in July 2007 [11].

### Study location and participants

Owing to itinerancy, the exact number of inhabitants in Bugoigo is not precisely known but is likely in the region of several thousand. The village contains up to three thousand traditional hut dwellings which stretch 3–4 km along the lakeshore and up to 1–2 km inland. Sanitation and hygiene in this village is minimal with few potable water sources and insufficient pit latrines. Household water is typically drawn directly from the lake at specific collection points and then taken back to each homestead in plastic jerry cans for subsequent domestic use. These lakeshore margins, like elsewhere on Lake Albert, provide conducive aquatic habitats for *Biomphalaria* spp., the intermediate snail hosts of *S. mansoni*, and can be found throughout the year, although infected snails vary in numbers seasonally [28], [29].

The immediate and longer-term objectives of this study were explained to the local community mobiliser who identified a total of 134 mothers that were willing to participate, bringing up to two of their infants/preschool children (≤5 years of age), and attend the two-day clinic commencing on the following day. After obtaining written informed consent from each mother on her own behalf and on behalf of her child(ren), urine, stool and fingerprick blood samples were obtained from all participants on the first day of the clinic. Mothers were then asked a suite of detailed questions recording their demography and water contact behaviours (the questionnaire is available upon request to the corresponding author).

After receipt of the second-day stool (and urine sample), all participants, regardless of their infection status, were treated for schistosomiasis and soil-transmitted helminthiasis with PZQ (40 mg/kg) (CIPLA, Mumbai, UK) and 400 mg albendazole (GSK, Uxbridge, UK) under medical supervision in conditions typical of mass-drug administration [30]. For smaller children, a chewable albendazole half-tablet (200 mg) was given and PZQ tablets were first crushed in orange juice before being administrated by spoon-feeding by their mother under supervision. The diagnostic findings for schistosomiasis here are reported for the children only.

### Schistosome antigens in urine (ADM)

Each child's urine sample was visually inspected for macro-haematuria/turbidity and a random sample was tested for micro-haematuria with Hemastix (Bayer, UK) to exclude the possibility of urinary schistosomiasis or other active urinary tract infections. A 50 µl aliquot was then tested for the presence of schistosome circulating cathodic antigen (CCA) using a commercially available lateral flow immuno-chromatographic urine dipstick (Rapid Medical Diagnostics, Pretoria, RSA) originally developed in Holland [31]. On a subset of 90 children, urine-CCA tests were performed in duplicate to assess variation between dipsticks.

To facilitate better recording of the visual intensity of the CCA reaction band within the test zone, results were visually graded against a reference chart for: trace, single (+), double (++) and triple (+++) positive reactions [32]. When creating binomial variables to depict infection status according to CCA, two variations were taken into account: the first considering trace results as negative infection status and the second considering trace results as positive infection status. The urine CCA reagent strip is referred to as an ADM (antigen detection method) from now on.

### Antibodies to soluble egg antigens (IEDM)

A commercially available ELISA kit (IVD Inc.; Carlsbad, USA) was used to test for host antibodies (IgG/M) to soluble egg antigens (SEA) according to manufacturer's instructions. Approximately 75 µl of finger-prick blood was taken from each child and serum was harvested, then diluted 1∶40 with specimen dilution buffer before loading a total of 100 µl into each ELISA microwell [11]. Positive and negative control sera were included on each batch of testing. Upon completion, each ELISA plate was placed on a white card and the colour within each microwell (ranging from colourless to yellow) was recorded by visual inspection. Positive reactions were classified either as trace (faint yellow), single (+, light yellow), double (++, yellow) or triple (+++, dark yellow) upon visual comparison with the control sera. The SEA-ELISA is referred to as an IEDM (indirect egg detection method) from now on.

### Direct egg-detection methods in stool (DEDM)

Three parasitological methods Kato-Katz, percoll and FLOTAC, henceforth referred to as direct egg detection methods (DEDMs), were attempted on each stool specimen to visualise eggs. However, owing to the differing amounts of stool required for each technique, it was not always possible to assemble a complete data set for every child with each of these three methods.

Duplicate Kato-Katz (K-K) thick smears (41.7mg) were made from first and second day stool samples (*N = *242 children) [33]. The four faecal smears were each examined under the microscope at x100, schistosome eggs were counted and later expressed as eggs per gram (epg) of faeces. Infection intensity was classified as light (1–100 epg), medium (101–400 epg) and heavy (>400 epg) infections according to WHO guidelines [5]. The methodology of Eberl [34] using sedimentation of schistosome eggs by centrifugation through a solution of percoll (Percoll 77237 (1.130 g/ml), Fluka, Sigma-Aldrich Chemie GmbH, Switzerland) was also implemented on-site to visualize eggs (*N = *96 children on first day stool). The egg-floatation procedure known as FLOTAC [35] was performed off-site back in Kampala on a formalin-fixed stool specimen archive (*N = *191 children taken from the second day stool) whereby schistosome eggs are collected by floatation centrifugation through a solution of zinc sulphate at specific gravity of 1.35.

### Data handling and statistical analyses

Data were collected from each individual using pro-forma data sheets, which were then transferred into electronic format using Microsoft Excel. The data thus collated were analysed using MS Excel and R statistical package version 2.8.0 [36]. For prevalence data and diagnostic parameters, 95% confidence intervals (CI_95_) were estimated using the exact method [37]. Prevalence comparisons were performed using (one-tailed) Fisher's exact modification of the 2×2 chi-squared test [38]. For infection intensity values, the arithmetic mean of positive cases was chosen as the measure of central tendency. Data from the FLOTAC and percoll methods were analysed by combining with K-K results and revising the diagnostic criterion so individuals were considered positive if an egg was detected by at least one DEDM.

The diagnostic performances of the ADM (including and excluding trace reactions as a positive diagnosis) and IEDM were tested qualitatively as a rapid diagnostic for intestinal schistosomiasis, considering DEDMs as the ‘gold-standard’. Additionally, a third ‘gold standard’ was created using data from the ADM (including and excluding trace reactions as positive diagnoses) against which to test IEDM data (*N = *242). Diagnostic sensitivity, specificity, positive predictive value (PPV) and negative predictive value (NPV) were calculated according to the different ‘gold standards’ [38]. The diagnostic powers of ADM and IEDM were calculated using all individuals, and then segregated by sex or age (≤3 years of age versus >3 years of age). *P*-values <0.05 were considered indicative of statistical significance [38].

### Ethical approvals

Approvals for this study were granted by the Ugandan Council for Science and Technology and the London School of Hygiene and Tropical Medicine (application numbers 06.45 and 5538.09). After sensitisation of the local community to the study objectives, verbal assent was first requested from each mother which was then formalised upon written informed consent (for her and behalf of her child), as either a thumbprint or signature on data recording sheet. This was witnessed by a Vector Control Division Officer. PZQ treatment (40 mg/kg) was offered to all study participants irrespective of their infection status.

## Results

A complete data set for the ADM, IEDM and Kato-Katz examinations was obtained from a total of 242 children (134 boys: 108 girls, mean age 2.9 years, minimum 5 months and maximum 5 years). However, owing to insufficient amounts of stool available, the FLOTAC and percoll methods could only be performed on 191 and 96 children, with the former and latter finding 4 and 2 additional egg-positive cases, respectively.

### Estimating infection prevalence

**Figure 1:**
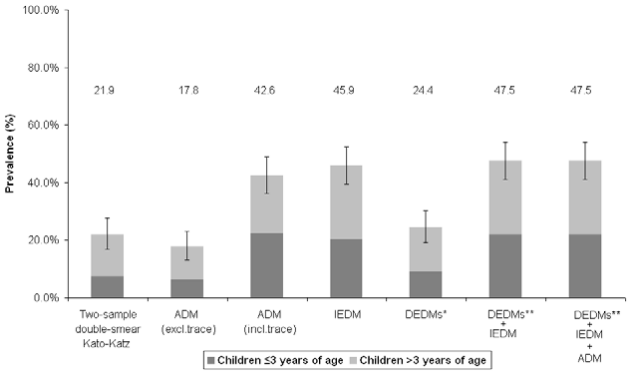
Barchart of the prevalence of intestinal schistosomiasis by each diagnostic technique (as well as pooling techniques, DEDM* representing results obtained from Kato-Katz, Percoll and FLOTAC methods) for the examined children. Note that the DEDMs under estimate infection prevalence and the addition of ADM to DEDMs/IEDM data did not further increase cumulative infection prevalence.

The prevalence of intestinal schistosomiasis estimated by each diagnostic method, and combinations thereof, is shown in Table 1 and Fig. 1. Prevalence inferred by DEDM, ADM (including trace reactions as positive diagnoses) and IEDM (considering traces as negatives) were: 24.4%, 42.6% and 45.9%. Of the children who were egg-positive by K-K, three quarters had ‘light’ intensity infections. Girls were equally as likely as boys to be diagnosed positively for intestinal schistosomiasis by ADM (Odds Ratio (OR)  = 1.06, p = 0.90) and DEDM examinations (OR = 0.72, p = 0.29). Children under the age of three, however, were less likely to be positive by ADM (OR = 0.51, p = 0.016) or by DEDM (OR = 0.26, p<0.0001) than their older counterparts. The prevalence of positives by IEDM was 45.9%. General prevalence inferred by pooling DEDM and IEDM was 47.7%, with no further change in prevalence when ADM was then added, see Fig. 1. There was no discordance between duplicate CCA testing for negative or positive classifications (data not shown).

**Table 1:** Prevalence and intensity of intestinal schistosomiasis and age of first positive by each diagnostic method.

	No. tested	Prevalence (%)	CI_95_ (%)	Mean epg (of positives)	AFP$
*DEDM (Two-sample double-smear Kato-Katz)*	242	21.9	16.9–27.6	86.4	9 months
* KK light intensity (<100 epg)*		16.9	12.4–22.3		9 months
* KK medium intensity (100*–*399 epg)*		4.5	2.3–8.0		3 years
* KK heavy intensity (>399 epg)*		0.4	0.0–2.3		5 years
*ADM (CCA incl. trace)*	242	42.6	36.3–49.1	NA	6 months
* + reaction*		17.8	13.2–23.2		9 months
* ++ reaction*		11.6	7.8–16.3		11 months
* +++ reaction*		1.7	0.5–4.2		2 years
*IEDM (SEA-ELISA)*	242	45.9	39.5–52.4	NA	6 months
* + reaction*		15.3	11.0–20.5		6 months
* ++ reaction*		19.4	14.6–25.0		1 year
* +++ reaction*		11.2	7.5–15.8		9 months
*DEDMs (all* * *)*	242	24.4	19.1–30.3	NA	NA
*IEDM+DEDM +ADM*	242	47.5	41.1–54.0	NA	NA

$ Age of first positive.

*Note not all 242 children were examined with percoll and FLOTAC (see methodology).

### Ages of becoming first positive

The age of first positive (AFP) for each method is presented in Table 1. For DEDM, the youngest child with eggs in stool was 9 months old, with medium and heavy infections found at 3 and 5 years of age, respectively. For ADM, trace reactions, single, double and triple positives were found in an ascending series of 6 months, 9 months, 11 months and 2 years of age, respectively. For IEDM, trace reactions began at 5 months of age while single, double and triple positive reactions were found in children as young as 6 months, 1 year and 9 months old, respectively. All tests concur on a mean age of first infection within the third year of life. ADM detected infections slightly ahead of I/DEDMs (3.2 years v. 3.4 years v. 3.7 years, respectively). The order of this temporal series is largely concordant with an absolute minimum age of becoming first positive.

### Cross-tabulations of diagnostic scores

In the absence of a genuine ‘gold standard’ where the infection status of each child is precisely known, it is necessary to explore relationships between diagnostic scores and infection intensities empirically, and to cross-tabulate diagnostic permutations by investigation. There was negligible variation in diagnostic performance of all protocols tested when classifying the data according to sex and age (data not shown) and general trends were reported from now on.

**Figure 2:**
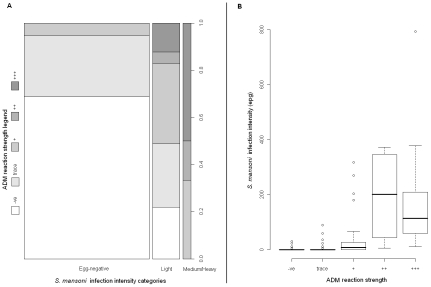
Comparisons of ADM and DEDM. **Figure 2A** Rectangular bar chart representing egg infection intensity classifications (as calculated by Kato-Katz) versus reaction intensity of the ADM (visual strength of the CCA urine dipstick test band). **Figure 2B** Boxplot of the egg faecal epg against ADM reaction intensity shows a positive increasing association.

Plotting the relationship between ADM and DEDM revealed some immediate trends, Fig. 2. Whilst there were children positive for ADM who were egg-negative, as the epg increases there was a corresponding increase in the proportion of positive ADM tests and once medium/heavy intensity infections were reached, all ADM tests were clearly positives, see Fig. 2A. Plotting the faecal epg of each child against the intensity of the corresponding ADM test further revealed this positive association, see Fig. 2B.

**Figure 3:**
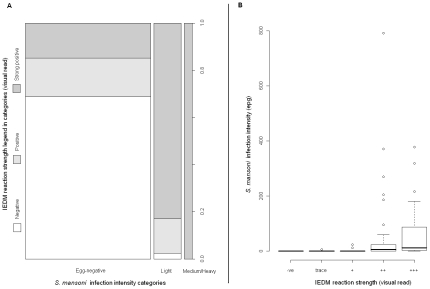
Comparisons of IEDM and DEDM. **Figure 3A** Rectangular bar chart representing egg infection intensity classifications (as calculated by Kato-Katz) versus reaction intensity of the IEDM (visual strength of the SEA-ELISA test well). **Figure 3B** Boxplot of the egg faecal epg against IEDM reaction intensity shows a positive increasing association.

Considering the relationship between IEDM and DEDM revealed similar trends, see Fig. 3. Despite some children being positive for IEDM while being egg-negative, as the faecal epg increases there was a corresponding increase in the IEDM reaction strength, with all medium/heavy intensity infections diagnosed as clear strong positives (Fig. 3A & B).

**Figure 4:**
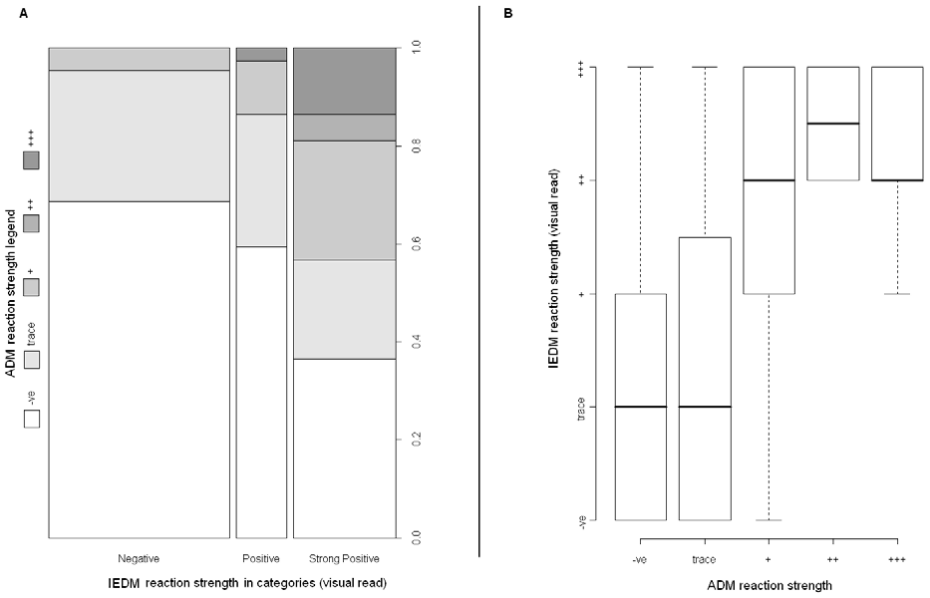
Comparisons of ADM and IEDM. **Figure 4A** Rectangular bar chart representing ADM intensity classifications (visual strength of the CCA urine dipstick test band) versus reaction intensity of the IEDM (visual strength of the SEA-ELISA test well). **Figure 4B** Boxplot of the IEDM reaction intensity against ADM shows a positive increasing association but the relationship is less clear-cut than that shown in **Figures 2B** & **3B**.

The relationship between ADM and IDEM was less clear-cut. Children who were ADM negative or trace had a median negative (or trace) IEDM reaction, but the proportionate increase of ADM positives with rising IEDM designations of positive (+) or strong positives (++/+++) was not as great as that seen with DEDM. For example, nearly 40% of children who were IEDM strong positive elicited a negative ADM reaction, Fig. 4A. As the intensity of the ADM result stepped up towards double and triple positive reactions, this typically corresponded better with increasing IEDM classifications, Fig. 4B.

Using available data it was possible to conduct an exploration of diagnostic performances of the ADM and IEDM versus DEDM and against each other (Table 2). First, considering an ADM trace reaction to be an infection negative and comparing with positive diagnosis by at least one of the DEDMs, the ADM had a sensitivity of 59.3%, specificity of 95.6%, PPV of 81.4% and NPV of 87.9%. When considering an ADM trace reaction to be a positive infection, and comparing to diagnosis by at least one of the DEDMs, ADM had a sensitivity of 81.4%, specificity of 69.9%, PPV of 46.6% and NPV of 92.1%.

**Table 2:** Comparison of diagnostic scores by ADM and IEDM using DEDM (all) as ‘gold standard’.

Diagnostic test	Diagnostic target	N	Sensitivity	Specificity	PPV	NPV
			(%,CI _95_)	(%,CI _95_)	(%,CI _95_)	(%,CI _95_)
*ADM (excl. trace)*	*DEDM*	242	59.3	95.6	81.4	87.9
			(45.8–71.9)	(91.6–98.1)	(66.6–91.6)	(82.6–92.1)
*ADM (incl. trace)*	*DEDM*	242	81.4	69.9	46.6	92.1
			(69.1–96.3)	(62.7–76.5	(36.7–56.7)	(86.3–96.0)
*IEDM*	*DEDM*	242	93.2	69.4	49.5	96.9
			(83.5–98.1)	(62.2–76.0)	(40.0–59.2)	(92.4–99.2)
	*ADM (excl. trace)*	242	86	62.8	33.3	95.4
			(72.1–94.7)	(56.0–69.5)	(24.7–42.9)	(90.3–98.3)
	*ADM (incl. trace)*	242	60.2	64.7	55.9	68.7
			(50.1–69.7)	(56.2–72.7)	(46.1–65.3)	(60.0–76.5)

The IEDM when compared to diagnosis by DEDM, demonstrated a sensitivity of 93.2%, specificity of 69.4%, PPV of 49.5% and NPV of 96.9%. For details on the performance of the ADM or IEDM compared to diagnosis by all DEDMs (using a subset of the data), and for CI_95_ around each value, see Table 2.

## Discussion

With limited access to safe water sources, and high levels of local transmission of *S. mansoni,* conditions in Bugoigo are particularly conducive for young children to acquire *S. mansoni* infections, and from a very early age. Approximately half of our children had intestinal schistosomiasis. As might be expected, regardless of techniques used, there was an obvious positive association between increasing diagnostic patency of infection with increasing age of the child. Presumably this was resultant from a progressive temporal accumulation of antigens, eggs and antibodies. Congruence between diagnostic methods became most apparent in children between 3¼–3¾ years of age, broadly consistent with the overall mean age of infected children within our sample. Prevalence of intestinal schistosomiasis in children under 3 years of age, however, was 35.5% (CI_95_ 27.9–43.8%) and other studies have also revealed that schistosomiasis in very young children can be common [9], [11], [39].

While egg excretions of these children were of ‘light’ intensity, such infected children will not normally receive praziquantel treatment until they have either entered primary school or if the NCP now formally extends its treatment remit to include this ageclass. Thus an infected child could therefore wait up to 3–4 years before receiving first treatment, and with this may have already entered a more ‘chronic’ stage of disease [40], [41], [42], [43]. For example, earlier clinical and ultrasound studies in Uganda in children aged 6 and above, have shown significant hepatosplenomegaly (i.e. putative morbidity from intestinal schistosomiasis), and while they have not yet developed pipe-stem liver fibrosis, up to 15% can have diffusely echogenic livers with pocketed foci, typical of image pattern B (‘the starry sky’ classification) [3], [27]. Without medication, it is likely that these preschool children will progress towards ‘moderate’ infection intensities before they become of school age. This might better explain the observations of Balen *et al*. that many adolescent Ugandan children have surprisingly severe intestinal schistosomiasis [44]. Although it is not yet proven that infection in very early childhood leads to heightened morbidity in later childhood and adolescence, this scenario appears plausible.

From animal models, it is known that only a fraction of penetrating cercariae successfully migrate to, and later mature in, the hepatic portal system. After adult worms reach full fecundity, schistosome eggs can be found in stool around 6 weeks after cercarial exposure and it is commonly held that females of *S. mansoni* produce up to 100–300 eggs per day, although many fail to be voided into the faeces [16]. Given the insensitivity of DEDMs in stool [34], it is not surprising that false negatives are inferred and the low egg-detection threshold(s) likely contribute to the longer apparent lag of 7–8 months between infection and egg-patency apparent between experimental schistosomiasis and the situation in the field. Moreover, it should be noted that the relationship between excreted eggs in stool and worm burdens is not always straightforward [45] and that infected laboratory animals are typically exposed with a single substantive dosing of cercariae. By contrast, and in this natural setting, exposure and infection is likely a more gradual process, i.e. the so-called trickle infection dynamic [46], and our children are at least two orders of magnitude greater in body size than most animal models.

### An age of first infection?

While some children were patently infected during the first year of life, others were not. Thus a sub-set of children exists with increased infection risk factors which we explain by the following synopsis. As children are born throughout the year, in a largely asynchronous fashion, whilst their initial age of first exposure to unsafe water might be broadly similar (i.e. within first few months of life as mothers begin to bathe them in jerry-can collected water or in the lake directly) their accumulated infection risk will not be equivalent owing the seasonality of local transmission factors and their particular timeframe of exposure within it contingent upon their mother's infant bathing and domestic water drawing practices [11].

Estimating this accumulated risk of infection reliably over the seasonal time frame of potential exposure is problematic as day-to-day variations within water collection times, its storage and actual domestic use (within each household) introduce many stochastic processes. Estimating cumulative infection risk is therefore easily confounded but an *ad hoc* investigation of infection risk associated with jerry-can collected water in June 2009, however, has confirmed that sentinel laboratory-bred mice could become infected to freshly drawn water [28]. Seasonal patterns, which operate in umbrella fashion over and above these specific-exposure patterns, no doubt effect this asynchronous age of first infection. Thus there will be no ‘absolute age’ of first infection but rather a ‘range of ages’ depending upon these intricate covariates of exposure. Only after a child has passed through sufficient ‘windows of exposure’, their probability of infection rises to an eventual certainty, after which, it is incumbent on the diagnostic tools to capture their parasitological status as accurately as possible.

### Comparison of diagnostic scores

From first appearances the ADM looks to best capture and identify infections in early stage, especially when we consider trace results as putative infection positives. A contentious issue in the use of the CCA reagent strip has been the interpretation of the exact diagnosis of this ‘trace’ result which can be confounded by non-specific inflammatory factors or breast-feeding [32], [47]. Interpretation of ‘trace’ is more contentious when surveying children under three years of age, where worm burdens are presumably lower than what might be expected in their school-aged counterparts. Interestingly, the percentage change in prevalence estimated according to the CCA reagent strip when excluding and including trace results as a positive diagnosis is significantly larger in the very young children (≤3 years of age) –10.1% v. 36.2% (+358%) – than in those aged four and five years of age –30.1% v. 52.7% (+175%) which is fitting with our understanding of increasing worm burdens through time. Thus we postulate that using ‘trace’ as positive firmly points towards a future use of the urine CCA-dipstick as an early indicator of infections which are as yet to become egg- or antibody-patent. It is particularly notable that the prevalence based on the ADM, when considering trace as positive, is very close to that of IEDM (Fig. 1), yet the diagnostic performance with it was not particularly congruent (see Fig. 3 and Table 2) so we still have an incomplete understanding of this infection progression. The dynamics of other ADM have been explored elsewhere in the context of recently acquired infection but not in very young children [48].

The ADM showed very promising diagnostic performance and robust field performance with high sensitivity and NPV scores (83.9% and 85.3%, respectively) when we considered trace results as a positive diagnoses and very high specificity and PPV scores (95.5% and 90.5%, respectively) when we considered trace results as negative diagnoses. This bimodal use of the test criteria could be advantageous from a control perspective. For instance, if a confident estimate of the suspected occurrence of infections within a population is needed, one should consider trace results as positives. On the other hand, to monitor the prevalence of ‘actual’ infection, or rather more easily identify those who do not, one should consider trace results as negatives. The former would be important if treatments were to be given out *en masse* as triggered by exceeding an aggregated local prevalence threshold while the latter would be important if treatment were to be withheld in an individual patient setting on the basis of test and treat.

### Towards promotion of safe water

With the gradual rise of infection prevalence in older children (over and above our asynchronous infection hypothesis), this trend must represent the spread of several risk factors, rather incipiently, across our cohort. Aggregation of infections in schistosomiasis is well-known [49] but it would be interesting to establish why approximately half of our study cohort had no evidence of infection despite living within the same village. As we were insufficiently aware of the exact locations of sampled households within the village, this could simply represent a cryptic spatial micro-patterning (i.e. these children who live slightly further away from the lake have less contact with viable cercariae) so we are now undertaking fine scale mapping of these individual households with GPS units. If, however, other causal factors could be identified and, perhaps more importantly, were these amenable to manipulation, it could lead to future infection mitigation measures.

Presently, within the NCP there are no health education materials targeted towards these mothers and their young children. More importantly and in terms of policy realignment of the NCP, a useful formal recommendation would be to initiate cross-sectorial activities with water and sanitation NGOs to improve immediately the domestic water quality at Bugoigo and elsewhere along the Lake Albert shoreline. Rather than focusing upon expensive infrastructure development, it could be achieved by introduction of simple water storage or modification measures. For example, as schistosome cercariae are an ephemeral larval stage, freshly drawn water can be rendered harmless for schistosomiasis by simple resting for 24 hrs, by crude filtration or by introduction of mild disinfectants [16]. Thus without initiating a better dialogue with these women of children bearing age through better public health education, mothers will remain sadly ignorant of the risks that making use of this unsafe water has for themselves and that of the future health of their child [11]. In this dialogue, the NCP should be receptive to explore which infection mitigation measures are best feasible and, by this token, help to provide safe water for domestic use which is well-received, implementable and effective.

### Epidemiological indicators and treatment needs

It is evident that infants and preschool children in Bugoigo, and other similar lakeshore villages of Uganda [12], are living in need of treatment. However, addressing how these children could be best identified is not yet clear, as are epidemiological parameters which should be collected for estimating treatment needs and also impact assessment. For example, should mass-treatment of all infants/preschool children take place when a sub-sample of an equivalent age range has been proven to be infected, or should treatment be allocated based at an individual level using the result of a diagnostic test in a ‘test and treat’ setting?

It is outside the remit of this present paper to make a cost-effectiveness calculation but a clear drawback of the IEDM method is that, whilst initially useful to establish if a child is infected (and there is no evidence in these data to suggest a passive maternal transfer of antibodies has been confounding), monitoring this parameter after treatment will be largely uninformative owing to residual antibody titres remaining after infection has putatively cleared. Thus IEDM is only useful at baseline but as an initial estimate of infection prevalence could be powerfully applied in identification and selection of villages, or sentinel locations, to first define the extent of the problem at intervention baseline. This of course assumes the majority of examined children can mount an antibody response and is not confounded by high levels of immune-suppression, by HIV for example, which is likely high in these fishing villages.

In contrast, both ADM and DEDMs have the potential ability to better track the dynamics of worm populations after treatment [22], [34] but the insensitivity of DEDMs is of particular concern. Put simply, numerous adult worms may reside within the host but are yet not depositing sufficient eggs to be visualised in stool on the day of sampling. Thus through lack of alternatives a pragmatic way forward would be to focus upon more widespread application of the ADM. The advantages of the ADM have been discussed elsewhere in the context of programmatic monitoring [14] but the future challenge will be for the NCP to meet the financial costs of using these rapid diagnostic tests in scale-up of operations. This is particularly true if these are to be used in a ‘test and treat’ setting when large numbers of tests would be utilized [14]. Given the low price of PZQ treatment, to maintain an affordable diagnosis versus treatment differential, a rational strategy would be to examine a sub-set of children and if local prevalence exceeded a given threshold, mass-treatment is advised. Such a strategy is presently within the resources available to the NCP but best sample sizes and prevalence thresholds remain to be determined.

## Supporting Information

Checklist S1 STROBE Checklist
(0.10 MB DOC)
