# Correction: *Schistosoma mansoni* Infections in Young Children: When Are Schistosome Antigens in Urine, Eggs in Stool and Antibodies to Eggs First Detectable?

**DOI:** 10.1371/annotation/1ee66788-2f23-4778-97dc-98a58030edd5

**Published:** 2012-10-03

**Authors:** J. Russell Stothard, Jose Carlos de Sousa-Figuereido, Martha Betson, Moses Adriko, Moses Arinaitwe, Candia Rowell, Fred Besiyge, Narcis B. Kabatereine

The second author's name was misspelled. The correct spelling is Jose Carlos de Sousa-Figueiredo. The correct citation is: Stothard JR, Sousa-Figueiredo JC, Betson M, Adriko M, Arinaitwe M, et al. (2011) Schistosoma mansoni Infections in Young Children: When Are Schistosome Antigens in Urine, Eggs in Stool and Antibodies to Eggs First Detectable? PLoS Negl Trop Dis 5(1): e938. doi:10.1371/journal.pntd.0000938

